# Socioeconomic inequalities in physical activity in Brazil: a pooled cross-sectional analysis from 2013 to 2019

**DOI:** 10.1186/s12939-021-01533-z

**Published:** 2021-08-23

**Authors:** Gerson Ferrari, Pedro Toteff Dulgheroff, Rafael M. Claro, Leandro F. M. Rezende, Catarina Machado Azeredo

**Affiliations:** 1 Escuela de Ciencias de la Actividad Física, El Deporte Y La Salud, Universidad de Santiago de Chile (USACH), Las Sophoras 175, Estación Central, Santiago Chile; 2 Pós-Graduação Em Saúde da Família, Faculdade de Medicina, Universidade Federal de Uberlândia, Uberlândia, Brazil; 3 Departamento de Nutrição, Escola de Enfermagem, Universidade Federal de Minas Gerais, Belo Horizonte, Brazil; 4 Departamento de Medicina Preventiva, Escola Paulista de Medicina, Universidade Federal de São Paulo, São Paulo, Brazil

**Keywords:** Inequality, Physical activity, Leisure, Commuting, Adults

## Abstract

**Background:**

Information on socioeconomic inequalities in physical activity over time is sparse in low- to middle-income countries. In this study, we examined trends in physical activity educational inequalities in adults from Brazil between 2013 and 2019.

**Methods:**

We analyzed data from seven cross-sectional studies including 359,038 adults (≥ 18 years) from the VIGITEL study conducted annually from 2013 to 2019. Participants responded to a questionnaire about physical activity (total, leisure, and commuting). Educational inequalities by sex and skin color were assessed through absolute (slope index of inequality – SII) and relative measures of inequality (concentration index – CIX).

**Results:**

We found large absolute and relative inequalities for leisure-time physical activity, favoring those with higher educational attainment (SII_2019_ = 35.4; CIX_2019_ = 11.82). Active commuting was more prevalent in intermediate education groups, with a slight inequality toward the less educated group (SII_2019_ = -2.8; CIX_2019_ = -0.4). From 2013 to 2019, the absolute educational inequality in physical activity (total, leisure, and commuting) remained unchanged; however, the relative inequality gap narrowed for total physical activity (CIX: 8.4 in 2013 to 5.5 in 2019) and leisure-time physical activity (CIX: 18.3 in 2013 to 11.8 in 2019). Educational inequality increased in leisure-time physical activity among women and non-white individuals, while it reduced among men and white individuals; for active commuting, inequality decreased among women, and increased among men and white individuals.

**Conclusions:**

Inequality in total physical activity and leisure-time physical activity favors the most educated groups in Brazil. Over time, relative educational inequality decreased for total and leisure-time physical activity, while no progress was found for absolute inequality.

## Introduction

Social determinants of health are responsible for the distribution of major risk factors for non-communicable diseases (NCDs), such as unhealthy food consumption, tobacco use, higher alcohol consumption, and low physical activity [1]. Studies monitoring socioeconomic inequalities in adult risk factors over time are important for health planning, but are sparce in low- and middle-income countries [2].

Brazil is one of the most unequal countries worldwide [2], even with the development of social programs in recent years with favorable impacts on adult health [3–6]. Importantly, in Brazil, as in most countries, socioeconomic differences in health are largely driven by differences in educational attainment [7].

Adults with lower education attainment tend to be in lower socioeconomic positions leading to worse health outcomes relative to those with higher education attainment, referred to as educational gradients in health. For instance, most studies in Brazil have found higher prevalence of physical inactivity among adults with lower education levels than among those with higher educational levels [8–11]. However, the extent of socioeconomic inequalities in adult physical activity and how it has changed over time in Brazil is uncertain.

Monitoring health inequality is essential to inform policies, programs, and practices to improve population health [12]. It is also important to evaluate the progress of health interventions that are designed and delivered with specific equity targets, such as the “*Academia da Cidade Program*” aimed to promote physical activity in Brazil in public spaces for free [13]. The reduction of inequality is a common goal. If certain population subgroups are less likely to engage in protective behaviors and suffer a disproportionate burden of morbidity, this endangers the well-being of a society at large and, in some situations, even holds back health progress for the most advantaged [12].

In this study, we examined educational inequality trends in physical activity among Brazilian adults between 2013 and 2019, using data from the Surveillance of Risk and Protection Factors for Chronic Diseases by Telephone Survey, the VIGITEL study.

## Methods

### Study design and source of data and sample

VIGITEL is a cross-sectional annual telephone survey (carried out since 2006) for monitoring health indicators of adults (aged ≥ 18 years) in 27 Brazilian capitals and the Federal District (DF) [14]. A sample of approximately 2000 individuals is interviewed in each city per year, so every indicator included in the system can be assessed with a 95% confidence interval (CI) and a sample error of 2 percentage points (pp). The sampling process is performed in two stages. The first consists of the sampling of 5000 landline telephones per city (from lists of valid household numbers provided by the main phone operators in the country), followed by reorganization in twenty-five replicas, each replica reproducing the same proportion of lines by postal code (ZIP code) of the original list. Each landline selected is contacted up to six times on different days and times (from 09.00 to 21.00 h, including weekends and holidays) to verify its eligibility. Non-residential lines, out-of-service lines, and lines that do not answer to any attempt of contact are considered ineligible. At the second stage, one adult among the residents of each household is randomly selected and invited to participate in the survey. Weighting factors provided by the Ministry of Health equate the distribution of the population interviewed by VIGITEL with that predicted for the entire adult population of each city. The weighting factors are estimated in two steps. The first, aims to correct the unequal probability of selection of households with more than one landline telephone or with more than one resident; and the second, to equate the distribution of the population interviewed in each city (by sex, age, and schooling) to its entire population (based on the official projections for each year, through Rake procedure) [14].

Our initial analytical sample included 359,038 participants interviewed between 2013 and 2019. For the analysis of subgroups by skin color, those who did not declared it were excluded (*n* = 16,266; 4,53%), comprising 342,772 interviews.

### Assessment of physical activity

Participants were asked to report the average time they spent per week in leisure-time physical activity (LTPA), commuting, and occupational activities [14]. For LTPA, participants were also asked about types of exercise and sports practiced. Participants who reported a minimum of 150 min/week of moderate intensity activities (walking, treadmill walking, bodybuilding, hydrogmnastics, swimming, martial arts, fights, cycling, volleyball, foot volley, or dance) or 75 min/week of vigorous intensity activities (running, treadmill running, aerobic gymnastics, futsal, soccer, basketball, or tennis) were considered physically active in the LTPA domain. Active commuting included walking or cycling to or from school/work, considering commuters to be physically active if they reported at least 30 min a day. As no specific recommendations for leisure and active commuting are available, the thresholds used periodically used in the VIGITEL for monitoring purposes were employed in the present study. For occupational physical activities, participants were asked about weekly frequency and duration of activities involving carrying weight or walking for a long time, during work. Total physical activity was calculated by summarizing LTPA, commuting and occupational activities. Participants were considered physically active if they reported a minimum of 150 min of moderate intensity activities or 75 min of vigorous intensity activities per week, or an equivalent combination of both intensities [15].

### Equity stratifiers

Participants were asked to indicate educational attainment in levels (e.g., primary, high-school, college, and postgraduation) and years of completion (e.g., 1, 2, and 3 years of high school), which was converted into number of years of study (0–3, 4–8, 9–11, and ≥ 12 years of study), as well as their perceived skin color (white and non-white; the latter included the following categories: black, brown, yellow, or indigenous) and their sex (women and men) [14].

### Statistical analysis

Description of participants’ characteristics according to survey years was presented as means and proportions. Temporal trends were formally tested using linear regression including survey years as an independent variable.

Annual prevalence of LTPA, active commuting, and total physical activity were described according to sex, number of years of study, and self-reported skin color. We also calculated the proportion of LTPA and commuting activities to total physical activity by number of years of study.

Complex measures of inequality such as the slope index of inequality (SII) and the concentration index (CIX) were used to estimate educational inequalities in physical activity. Both SII and CIX consider all levels of education attainment to compare physical activity levels. We estimated the SII using logistic regression to avoid predicting implausible values below zero or above one, considering that physical activity domains were presented as proportions [16]. To facilitate their visualization in tables and graphs, the results of SII and CIX were multiplied by 100. Results equal to zero represent total equality and results equal to ± 100, total inequality. Negative values indicate a higher prevalence in the least educated groups. CIX values lower than -20 or greater than 20 are considered relevant indicators of inequality [12]. Subsequently, the SII and CIX data were stratified by sex and skin color.

We also used absolute and relative population attributable risk. The first one indicates the possible improvement (in percentage points) of gap in physical activity if all subgroups had the same level of physical activity of the most favored group (herein considered ≥ 12 years of study). The relative measure of population attributable risk represents the possible proportional improvement if there was no inequity between subgroups, and it is obtained dividing the absolute population attributable risk by the overall prevalence in the total population. The greater the result, the greater the inequality [12]. Negative results represent that promoting equality between educational groups would reduce the average population level of a given physical activity indicator.

Linear regression using variance-weighted least squares was performed in SII and CIX to assess changes over time (2013–2019) [17]. Statistical analyses were performed using Stata/SE® 15.1 software.

### Ethical aspects

The National Research Ethics Commission approved VIGITEL (processes nº 13.081/2008, 355.590/2013, and 2,100,213/2017), CAAE: 65,610,017.1.0000.0008. The database is available at: http://svs.aids.gov.br/download/Vigitel/, and it does not allow identification of participants.

## Results

Participant characteristics by survey years are presented in Table 1. Between 2013 and 2019, women represented 54.0% of sample. The participants mean age was 42.2 years, ranging from 41.5 years in 2013 to 42.7 years in 2019. Over time, the proportion of individuals with 12 or more years of education increased from 25.9% to 32.8%, as well as the proportion of non-white participants (50.0% to 58.4%). There was also an increase in the prevalence of adults achieving 150 min/week of total physical activity (50.6% to 55.2%) and LTPA (33.8% to 39.0%), and 30 min/day of active commuting (12.0% to 14.1%).

**Table 1:** Sociodemographic characteristics and physical activity indicators, according to survey year of VIGITEL (2013–2019)

Characteristics	Survey year						
	2013	2014	2015	2016	2017	2018	2019
Individuals (n)	52,929	40,853	54,174	53,210	53,034	52,395	52,443
Sex (%), women	53.9	53.9	53.9	54.0	54.0	54.0	54.0
Mean age (years)*	41.5	41.7	42.0	42.2	42.4	42.6	42.7
Education (in years of study)							
4 years*	7.6	7.1	6.8	6.7	6.1	6.1	6.3
8 years*	29.0	28.8	27.8	25.8	24.7	24.1	22.5
9–11 years	37.5	38.1	38.1	35.9	37.3	37.9	38.4
12 or more*	25.9	25.9	27.3	31.6	31.9	31.8	32.8
Skin color (%), white	41.5	39.7	40.8	43.6	42.0	41.3	41.0
Non-white*	50.0	50.6	57.5	49.5	56.9	58.0	58.4
Non-declared*	8.5	9.7	1.7	6.9	1.0	0.6	0.6
Physically active^a^							
Total physical activity*	50.6	51.3	52.5	54.9	54.0	55.9	55.2
Leisure*	33.8	35.3	37.6	37.6	37.0	38.1	39.0
Active commuting*	12.0	12.3	11.9	14.4	13.4	14.4	14.1

^*^P_trend_ < 0,05

^a^We used the following thresholds for considering participants physically active in each of the physical activity indicators: total physical activity (summarizing LTPA, active commuting and occupation activities in min/week): 150 min of moderate intensity activities or 75 min of intense vigorous intensity activity activities per week; LTPA: of 150 min/week of moderate intensity or 75 min/week of vigorous intensity activities per week; Active commuting: at least 30 min a day of walking or cycling to or from school/work)

**Fig. 1:**
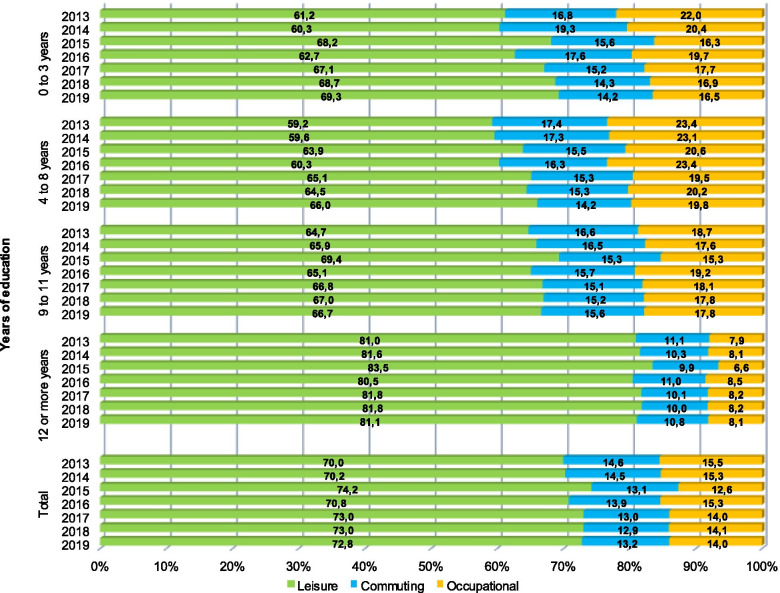
Proportion of leisure-time physical activity and active commuting to total physical activity according to the number of years of education. Footnote: We used the following thresholds for considering participants physically active in each of the physical activity indicators: total physical activity (summarizing LTPA, active commuting and occupation activities in min/week): 150 min of moderate intensity or 75 min of vigorous intensity activities per week; LTPA: 150 min/week of moderate intensity or 75 min/week of vigorous intensity activities; Active commuting: at least 30 min a day of walking or cycling to or from school/work)

Figure 1 displays the proportion of LTPA and commuting activities to total physical activity by education level from 2013 to 2019. The relative contribution of physical activity domains remained similar over time. LTPA was the domain with the highest relative contribution to total physical activity (70.0% in 2013; 72.8% in 2019), while active commuting (14.6% to 13.2%) contributed relatively less. The relative contribution of LTPA was higher among individuals with 12 or more years of study (81.1% in 2019) compared with those with 0 to 3 years (69.3% in 2019). Active commuting was more frequent among people who had from 9 to 11 years of study (15.6% in 2019).

**Fig. 2:**
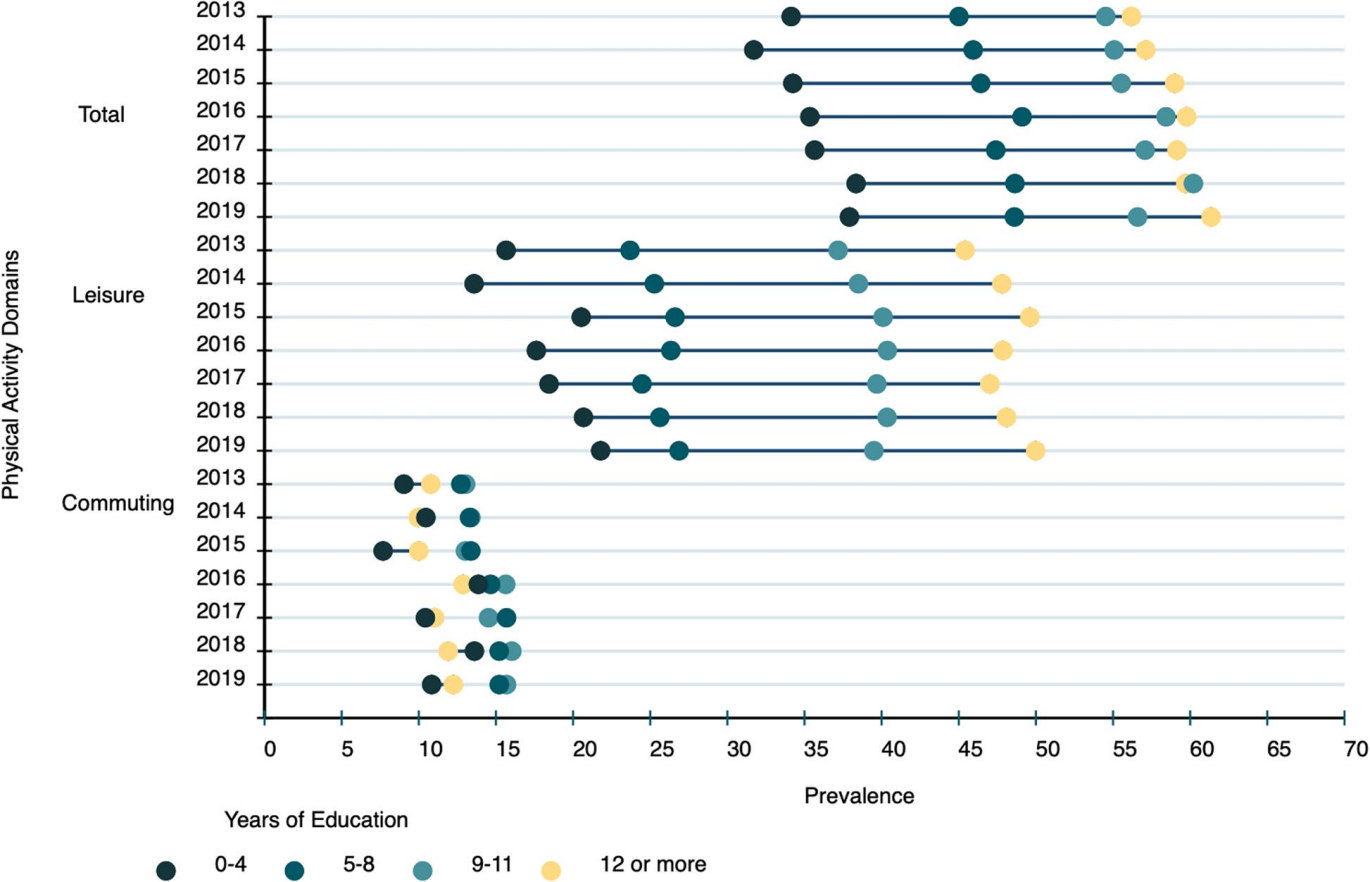
Time trends in total, leisure, and commuting physical activity by years of education in adults. Footnote: We used the following thresholds for considering participants physically active in each of the physical activity indicators: total physical activity (summarizing LTPA, active commuting and occupation activities in min/week): 150 min of moderate intensity activities or 75 min of intense vigorous intensity activity activities per week; LTPA: of 150 min/week of moderate intensity or 75 min/week of vigorous intensity activities; Active commuting: at least 30 min a day of walking or cycling to or from school/work)

Table 2 and Fig. 2 show the prevalence of total physical activity, LTPA and active commuting by education level, absolute (SII) and relative (CIX) inequality and absolute and relative population attributable risk in 2013 and 2019. In 2019, prevalence of ≥ 150 min/week of total physical activity and LTPA were higher in adults with ≥ 12 years of study (61.4% and 50.0%, respectively) than in adults with 0–4 years of study (37.9% and 21.8%). The highest prevalence of active commuting was observed in the subgroup with 9 to 11 years of education (15.7%). Both SII and CIX evidenced that total physical activity and LTPA were more prevalent among adults with higher educational level, while active commuting was more frequent among less educated subgroups. Absolute and relative population attributable risks were, respectively, 6.2 percentage points and 11.2% for total physical activity, 11.0 percentage points and 28.2% for LTPA, and -1.8 percentage points and -12.9 for active commuting. Overall, we observed a small reduction in the magnitude of the educational inequality for total physical activity and LTPA, especially for relative measures of inequality. For instance, CIX for total physical activity reduced from 8.4 in 2013 to 5.5 in 2019, whereas the reduction in CIX for LTPA reduced from 18.3 in 2013 to 11.8 in 2019. Absolute measures of educational inequalities in physical activity remained constant in the period (Table 2).

**Table 2:** Complex measures (percentage and 95% confidence intervals) of education inequality in total physical activity, leisure-time physical activity, and commuting physical activity. VIGITEL, 2013 and 2019

**Physical activity indicators**^a^	**Description of**			**Complex measures of inequality**		**Absolute population attributable risk (percentage points)**	**Relative population attributable risk (percentage)**
	**Complete Sample**	**Lowest education (0–4 years of study)**	**Highest education (≥ 12 years of study)**	**Slope Index of Inequality (SII)**	**Concentration Index (CIX)**		
**Total**							
2013	50.6 (49.8; 51.5)	34.1 (31.0; 37.4)	56.2 (54.6; 57.7)	21.7 (18.6; 24.8)	8.4 (7.4; 9.4)	5.6	11.1
2019	55.2 (54.3; 56.1)	37.9 (34.4; 41.5)	61.4 (59.8; 62.9)	22.5 (19.1; 26.0)	5.5 (4.5; 6.5)	6.2	11.2
**Leisure**							
2013	33.8 (33.0; 34.6)	15.7 (13.6; 17.9)	45.4 (43.8; 47.0)	34.8 (32.1; 37.5)	18.3 (17.0; 19.7)	11.6	34.3
2019	39.0 (38.0; 39.9)	21.8 (19.0; 24.8)	50.0 (48.3; 51.6)	35.4 (32.2; 38.7)	11.8 (10.5; 13.2)	11.0	28.2
**Commuting**							
2013	12.0 (11.5; 12.7)	9.0 (7.3; 11.1)	10.8 (9.7; 11.9)	-0.7 (-2.9; 1.4)	-0.8 (-3.6; 2.0)	-1.2	-10.0
2019	14.1 (13.4; 14.9)	10.8 (8.8; 13.3)	12.2 (11.1;13.5)	-2.8 (-5.4; -0.2)	-0.4 (-3.4; 2.5)	-1.8	-12.9

*Abbreviations*: *SII* Slope index of inequality, *CIX* Concentration index

^a^We used the following thresholds for considering participants physically active in each of the physical activity indicators: total physical activity (summarizing LTPA, active commuting, and occupation activities in min/week): 150 min of moderate intensity or 75 min of vigorous intensity activities per week; LTPA: of 150 min/week of moderate intensity or 75 min/week of vigorous intensity activities; Active commuting: at least 30 min a day of walking or cycling to or from school/work)

**Fig. 3:**
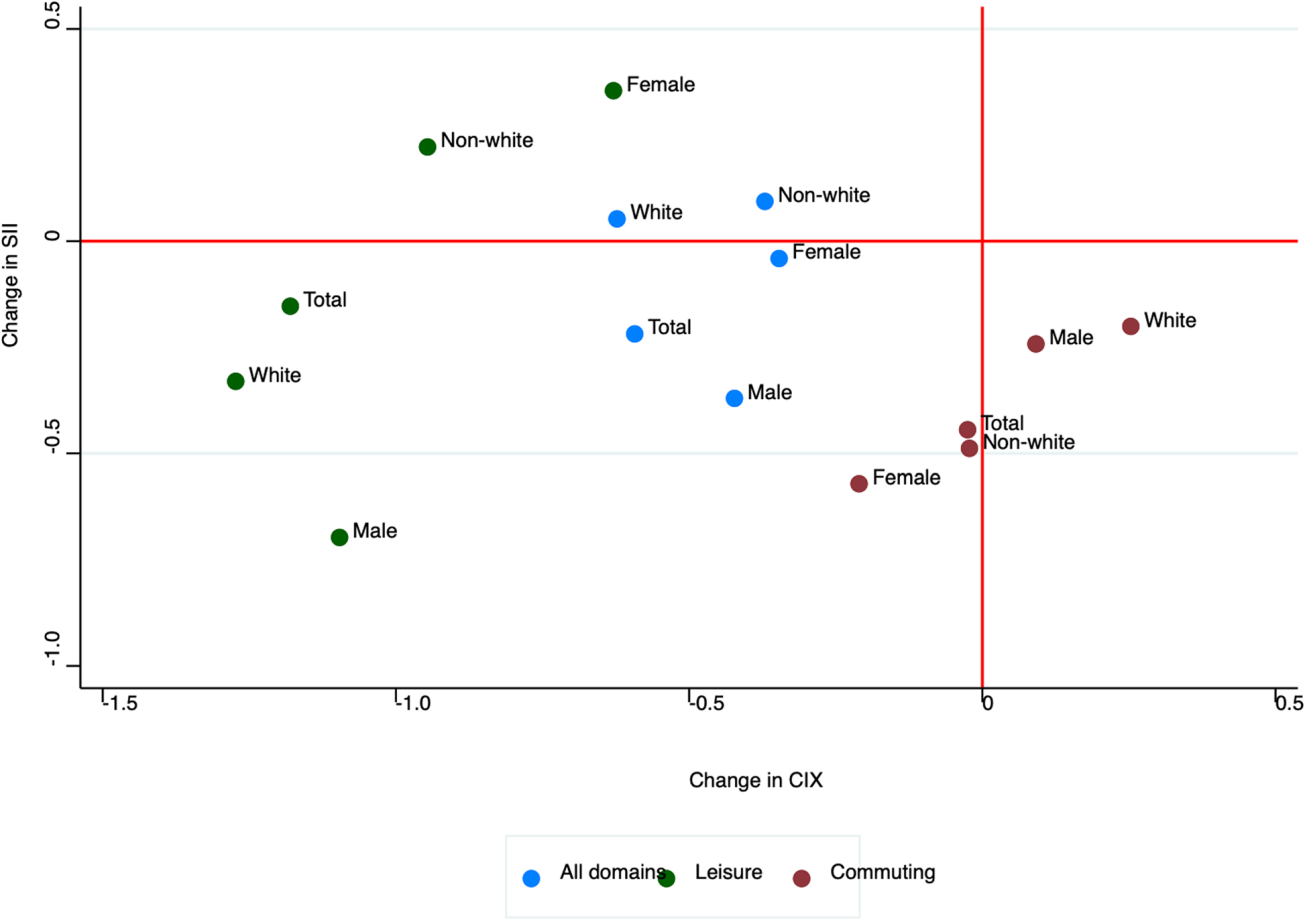
Difference between 2013 and 2019 in the slope index of inequality and concentration index of physical activity domains in adults. Footnote: We used the following thresholds for considering participants physically active in each of the physical activity indicators: total physical activity (summarizing LTPA, active commuting and occupation activities in min/week): 150 min of moderate intensity or 75 min of vigorous intensity activities per week; LTPA: of 150 min/week of moderate intensity or 75 min/week of vigorous intensity activities per week; Active commuting: at least 30 min a day of walking or cycling to or from school/work)

The absolute and relative educational inequality for total and leisure physical activity was, in general, higher among women than men, represented by positive and higher SII and CIX values. Over time, the absolute and relative educational inequality in total and LTPA decreased in all groups (total, women, men, white, non-white). This decrease was greater in men than in women, as well as in white individuals in relation to non-white individuals. There was no statistically significant decrease in educational inequality among subgroups for active commuting (Fig. 3).

## Discussion

In this comprehensive study assessing data from 359,038 Brazilian adults from 2013 to 2019, we found that relative educational inequality has narrowed for total and LTPA, although absolute educational inequality remained unchanged. For active commuting, there was an increase in absolute educational inequality. Total and LTPA were more prevalent in those with higher educational attainment, while active commuting was more prevalent among those less educated.

Total physical activity has increased in the Brazilian capitals over the last years, especially due to the increase in LTPA. LTPA is higher among groups of higher socioeconomic level. We also found that, over time, this (relative) difference in LTPA decreased between those with higher educational levels *vs* lower educational levels. These reductions in inequality were observed in all subgroups of sex and skin color; however, the reduction was greater in men than in women, as well as in white in comparison with non-whites individuals.

In 2011, the Ministry of Health launched the 2011–2022 Strategic Action Plan to Tackle Noncommunicable Diseases (NCDs) in Brazil aiming to promote the development and implementation of effective, integrated, sustainable, and evidence-based public policies for the prevention and control of NCDs [18]. The plan acknowledged social inequalities in LTPA and other lifestyle risk factors, and aimed to reduce them through surveillance of vulnerable groups and health care access for these groups [19]. For instance, the *Academia da Saúde* (Health Academy) is an example of health promotion strategy that uses public spaces equipped with infrastructure, equipment, and qualified professionals to promote the practice of physical activity free of charge, and therefore could potentially reduce health inequities [13]. There is a higher proportion of participants with low educational level participating in this program [13], which may explain the relative reduction in inequalities in LTPA over the last years. In general, LTPA increased in all educational groups; however, we observed a greater increase among those with lower education levels. Recent setbacks related to government funding raise concern about the continuity of this, so far, successful program.

We showed that active commuting was higher among the groups of intermediate educational level, without major changes over time. The determinants of active commuting are complex, usually not equally distributed among high and low socioeconomic neighborhoods [20, 21]. Factors such as safety related to crime and traffic accident rates, connectivity with parks and green areas [22], and the existence of cycle lanes, density, and availability of public transport are especially important for active commuting [20, 23, 24]. Cycle lanes and bicycle-sharing systems have been used to promote bicycling [25, 26]. In Brazil, bicycle-sharing systems expanded in the last decade through private partnerships promoted by municipalities [27, 28]. Bicycle-sharing users are more likely to be men, white, employees, younger, richer, and highly educated individuals, having unequal social and spatial distribution [29]. In Brazil, the mean income of the areas served by the bicycle-sharing systems is twice the cities’ mean income [28]. On the other hand, some studies found an inverse association between neighborhood socioeconomic position and active commuting [30, 31]. This could either be an indication that people with low socioeconomic position are more likely to engage in active commuting or, for example, that neighborhoods with a low socioeconomic position are more likely to make people engage in active commuting because of a higher density or more connectivity [32]. More research on this topic should be conducted to get a better insight into the determinants of active commuting [20]. So far, the main evidence supports that polices must focus on the promotion of walking and cycling infrastructure in poorest areas, considering specific needs of disadvantaged population [33].

Our study has several limitations. The sample of VIGITEL included adults living in Brazilian capitals and the federal district with access to landlines. Despite using weighting measures for the general population, we would expect some small differences in the prevalence of physical activity domains if we had included people without landline access and people from rural municipalities [34]. The access to landlines has reduced over time, and older and wealthier households are more likely to have and retain a landline in addition to a mobile phone [35]. Therefore, part of the changes observed in physical activity over time may be attributable to these changes in landline access (selection bias). Moreover, measurement error of physical activity is expected due to the use of questionnaires. VIGITEL does not collect data on household income, thus we relied on education level as our main indicator of socioeconomic inequalities. However, education level is a good proxy of socioeconomic level in Brazil, although education attainment also encompasses a potential for better health consciousness [36].

## Conclusion

Inequality in total and LTPA favors the most educated groups, while inequality in active commuting favors the least educated adults in Brazil. Over time, relative inequality decreased for total and LTPA, while no advances were found for absolute inequality. Among women and non-white groups, educational inequality widened for LTPA. Our results may be considered in future intervention and actions aimed at increasing population-wide physical activity and coping with non-communicable diseases.

## Data Availability

All datasets are available on each governmental website.

## Abbreviations

CIX: Concentration index
CI: Confidence interval
FD: Federal District
LTPA: Leisure-time physical activity
NCDs: Non-communicable diseases
PP: Percentage points
SII: Slope index of inequality
VIGITEL: Surveillance of Risk and Protection Factors for Chronic Diseases by Telephone Survey

## References

[1] Marmot M, Bell R (2019). Social determinants and non-communicable diseases: time for integrated action. BMJ.

[2] Solt F (2016). The standardized world income inequality database. Soc Sci Q.

[3] Landmann-Szwarcwald C, Macinko J (2016). A panorama of health inequalities in Brazil. Int J Equity Health.

[4] Martins AP, Monteiro CA (2016). Impact of the Bolsa Familia program on food availability of low-income Brazilian families: a quasi experimental study. BMC Public Health.

[5] Hone T, Rasella D, Barreto ML, Majeed A, Millett C (2017). Association between expansion of primary healthcare and racial inequalities in mortality amenable to primary care in Brazil: a national longitudinal analysis. PLoS Med.

[6] Rasella D, Harhay MO, Pamponet ML, Aquino R, Barreto ML (2014). Impact of primary health care on mortality from heart and cerebrovascular diseases in Brazil: a nationwide analysis of longitudinal data. BMJ.

[7] Duncan BB, Schmidt MI, Achutti AC, Polanczyk CA, Benia LR, Maia AA (1993). Socioeconomic distribution of noncommunicable disease risk factors in urban Brazil: the case of Porto Alegre. Bull Pan Am Health Organ.

[8] Ferrari GLM, Kovalskys I, Fisberg M, Gomez G, Rigotti A, Sanabria LYC, et al. Socio-demographic patterning of objectively measured physical activity and sedentary behaviours in eight Latin American countries: findings from the ELANS study. Eur J Sport Sci. 2019:1–12. 10.1080/17461391.2019.1678671. 10.1080/17461391.2019.1678671 31603392

[9] Werneck AO, Baldew SS, Miranda JJ, Diaz Arnesto O, Stubbs B, Silva DR (2019). Physical activity and sedentary behavior patterns and sociodemographic correlates in 116,982 adults from six South American countries: the South American physical activity and sedentary behavior network (SAPASEN). Int J Behav Nutr Phys Act.

[10] Peixoto SV, Mambrini JVM, Firmo JOA, Loyola Filho AI, Souza Junior PRB, Andrade FB (2018). Physical activity practice among older adults: results of the ELSI-Brazil. Rev Saude Publica.

[11] Cruz MSD, Bernal RTI, Claro RM (2018). Trends in leisure-time physical activity in Brazilian adults (2006–2016). Cad Saude Publica.

[12] World Health Organization (2013). Handbook on health inequality monitoring: with a special focus on low- and middle-income countries.

[13] Malta DC, Mielke GI, Costa NCP (2020). Pesquisas de avaliação do Programa Academia da Saúde.

[14] Ministério da Saúde. Secretaria de Vigilância em Saúde. Departamento de Análise em Saúde e Vigilância de Doenças Não Transmissíveis. Vigitel Brasil 2019: vigilância de fatores de risco e proteção para doenças crônicas por inquérito telefônico. Estimativas sobre frequência e distribuição sociodemográfica de fatores de risco e proteção para doenças crônicas nas capitais dos 26 estados brasileiros e no Distrito Federal em 2019. Brasília: Ministério da Saúde; 2020. p. 137.

[15] Bull FC, Al-Ansari SS, Biddle S, Borodulin K, Buman MP, Cardon G (2020). World Health Organization 2020 guidelines on physical activity and sedentary behaviour. Br J Sports Med.

[16] Barros AJ, Victora CG (2013). Measuring coverage in MNCH: determining and interpreting inequalities in coverage of maternal, newborn, and child health interventions. PLoS Med.

[17] Azeredo CM, de Rezende LFM, Mallinson PAC, Ricardo CZ, Kinra S, Levy RB (2019). Progress and setbacks in socioeconomic inequalities in adolescent health-related behaviours in Brazil: results from three cross-sectional surveys 2009–2015. BMJ Open.

[18] Ministério da Saúde. Secretaria de Vigilância em Saúde Departamento de Vigilância de Doenças e Agravos não Transmissíveis e Promoção da Saúde Relatório do III Fórum de Monitoramento do Plano de Ações Estratégicas para o Enfrentamento das Doenças Crônicas não Transmissíveis no Brasil. Brasília: Ministério da Saúde; 2018.

[19] Ministério da Saúde. Secretaria de Vigilância em Saúde Plano de ações estratégicas para o enfrentamento das doenças crônicas não transmissíveis (DCNT) no Brasil: 2011–2022. Brasília: Ministério da Saúde; 2011.

[20] Ferrari G, Werneck AO, da Silva DR, Kovalskys I, Gomez G, Rigotti A (2020). Is the perceived neighborhood built environment associated with domain-specific physical activity in Latin American adults? An eight-country observational study. Int J Behav Nutr Phys Act.

[21] Sallis JF, Cerin E, Kerr J, Adams MA, Sugiyama T, Christiansen LB (2020). Built environment, physical activity, and obesity: findings from the International Physical Activity and Environment Network (IPEN) adult study. Annu Rev Public Health.

[22] Le HTK, Buehler R, Hankey S (2018). Correlates of the built environment and active travel: evidence from 20 US metropolitan areas. Environ Health Perspect.

[23] Moran M, Van Cauwenberg J, Hercky-Linnewiel R, Cerin E, Deforche B, Plaut P (2014). Understanding the relationships between the physical environment and physical activity in older adults: a systematic review of qualitative studies. Int J Behav Nutr Phys Act.

[24] Foster S, Giles-Corti B (2008). The built environment, neighborhood crime and constrained physical activity: an exploration of inconsistent findings. Prev Med.

[25] Meddin R (2017). The bike-sharing world at the end of 2016. Bike-Shar. Blog.

[26] Sarmiento O, Torres A, Jacoby E, Pratt M, Schmid TL, Stierling G (2010). The Ciclovia-Recreativa: a mass-recreational program with public health potential. J Phys Act Health.

[27] Gauthier A, Hughes C, Kost C, Li S, Linke C, Lotshaw S (2013). The bike-share planning guide.

[28] Duran AC, Anaya-Boig E, Shakec JD, Garcia LMT, Rezende LFM, Sá TH (2018). Bicycle-sharing system socio-spatial inequalities in Brazil. J Transp Health.

[29] Woodcock J, Tainio M, Cheshire J, O’Brien O, Goodman A (2014). Health effects of the London bicycle sharing system: health impact modelling study. BMJ.

[30] van Lenthe FJ, Brug J, Mackenbach JP (2005). Neighbourhood inequalities in physical inactivity: the role of neighbourhood attractiveness, proximity to local facilities and safety in the Netherlands. Soc Sci Med.

[31] Van Dyck D, Cardon G, Deforche B, Sallis JF, Owen N, De Bourdeaudhuij I (2010). Neighborhood SES and walkability are related to physical activity behavior in Belgian adults. Prev Med.

[32] Rundle A, Diez Roux AV, Free LM, Miller D, Neckerman KM, Weiss CC (2007). The urban built environment and obesity in New York City: a multilevel analysis. Am J Health Promot.

[33] Gelius P, Messing S, Goodwin L, Schow D, Abu-Omar K (2020). What are effective policies for promoting physical activity? A systematic review of reviews. Prev Med Rep.

[34] Bernal RTI, Malta DC, Claro RM, Monteiro CA (2017). Effect of the inclusion of mobile phone interviews to Vigitel. Rev Saude Publica.

[35] Bernal RTI, Malta DC, de Araújo TS, da Silva NN (2013). Inquérito por telefone: pesos de pós-estratifi cação para corrigir vícios de baixa cobertura em Rio Branco. AC Rev Saúde Pública.

[36] Andrade CL, Szwarcwald CL, Gama SG, Leal MC (2004). Socioeconomic inequalities and low birth weight and perinatal mortality in Rio de Janeiro, Brazil. Cad Saude Publica.

